# Precision prediction of heart failure events in patients with dilated cardiomyopathy and mildly reduced ejection fraction using multi‐parametric cardiovascular magnetic resonance

**DOI:** 10.1002/ejhf.3425

**Published:** 2024-08-15

**Authors:** Daniel J. Hammersley, Srinjay Mukhopadhyay, Xiuyu Chen, Richard E. Jones, Aaraby Ragavan, Saad Javed, Husein Rajabali, Emmanuel Androulakis, Lara Curran, Lukas Mach, Zohya Khalique, Resham Baruah, Kaushik Guha, John Gregson, Shihua Zhao, Antonio De Marvao, Upasana Tayal, Amrit S. Lota, James S. Ware, Dudley J. Pennell, Sanjay K. Prasad, Brian P. Halliday

**Affiliations:** ^1^ King's College Hospital NHS Foundation Trust London UK; ^2^ National Heart and Lung Institute, Imperial College London London UK; ^3^ Royal Brompton & Harefield Hospital, Guy's & St Thomas' NHS Foundation Trust London UK; ^4^ Fuwai Hospital, State Key Laboratory of Cardiovascular Disease, National Center for Cardiovascular Diseases, Chinese Academy of Medical Sciences and Peking Union Medical College Beijing China; ^5^ Essex Cardiothoracic Centre Basildon UK; ^6^ Anglia Ruskin University Chelmsford UK; ^7^ Portsmouth Hospital University Trust Portsmouth UK; ^8^ London School of Hygiene and Tropical Medicine London UK; ^9^ British Heart Foundation Centre of Research Excellence, School of Cardiovascular and Metabolic Medicine and Sciences, King's College London London UK; ^10^ Department of Women and Children's Health King's College London London UK; ^11^ MRC Laboratory of Medical Sciences, Imperial College London London UK

**Keywords:** Heart failure, Mild dilated cardiomyopathy, Sudden cardiac death

## Abstract

**Aims:**

To assess whether left ventricular (LV) global longitudinal strain (GLS), derived from cardiovascular magnetic resonance (CMR), is associated with (i) progressive heart failure (HF), and (ii) sudden cardiac death (SCD) in patients with dilated cardiomyopathy with mildly reduced ejection fraction (DCMmrEF).

**Methods and results:**

We conducted a prospective observational cohort study of patients with DCM and LV ejection fraction (LVEF) ≥40% assessed by CMR, including feature‐tracking to assess LV GLS and late gadolinium enhancement (LGE). Long‐term adjudicated follow‐up included (i) HF hospitalization, LV assist device implantation or HF death, and (ii) SCD or aborted SCD (aSCD). Of 355 patients with DCMmrEF (median age 54 years [interquartile range 43–64], 216 men [60.8%], median LVEF 49% [46–54]) followed up for a median 7.8 years (5.2–9.4), 32 patients (9%) experienced HF events and 19 (5%) died suddenly or experienced aSCD. LV GLS was associated with HF events in a multivariable model when considered as either a continuous (per % hazard ratio [HR] 1.10, 95% confidence interval [CI] 1.00–1.21, *p* = 0.045) or dichotomized variable (LV GLS > −15.4%: HR 2.70, 95% CI 1.30–5.94, *p* = 0.008). LGE presence was not associated with HF events (HR 1.49, 95% CI 0.73–3.01, *p* = 0.270). Conversely, LV GLS was not associated with SCD/aSCD (per % HR 1.07, 95% CI 0.95–1.22, *p* = 0.257), whereas LGE presence was (HR 3.58, 95% CI 1.39–9.23, *p* = 0.008). LVEF was neither associated with HF events nor SCD/aSCD.

**Conclusion:**

Multi‐parametric CMR has utility for precision prognostic stratification of patients with DCMmrEF. LV GLS stratifies risk of progressive HF, while LGE stratifies SCD risk.

## Graphical abstract

In this study, 355 patients with dilated cardiomyopathy (DCM) and mildly reduced left ventricular ejection fraction (LVEF) were characterized using cardiovascular magnetic resonance (CMR), including feature‐tracking to calculate left ventricular global longitudinal strain (LV GLS) and late gadolinium enhancement (LGE). Over long‐term follow‐up, LV GLS > −15.4% was associated with a higher cumulative incidence of major heart failure (HF) events, whereas the presence LGE was associated with a higher cumulative incidence of sudden cardiac death (SCD) or aborted SCD (aSCD).

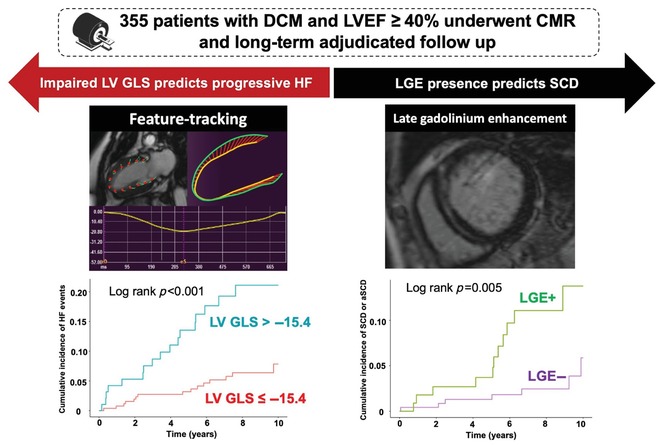

## Introduction

Dilated cardiomyopathy (DCM) is a major cause of heart failure (HF) globally. DCM‐associated morbidity and mortality principally results from pump failure or ventricular arrhythmias.^1^ Heterogeneous aetiology and variable response to therapy remain barriers to more precise risk prediction across the spectrum of phenotypic severity. Many patients with DCM have mildly reduced ejection fraction (DCMmrEF), either due to early disease detection or improvement in cardiac function from more severe phenotypes.^2^ Whilst those with DCMmrEF typically have fewer symptoms and generally good long‐term outcomes, a subset will go on to progressive HF or succumb to sudden cardiac death (SCD).^3^, ^4^ We have previously identified late gadolinium enhancement (LGE) on cardiovascular magnetic resonance (CMR) as an important determinant of SCD in this population.^5^ However, the incidence and predictors of progressive HF in patients with DCMmrEF remain unknown. It is also unclear whether the risk profile of patients with early disease is equivalent to those with improved cardiac function, recognizing their comparable level of mild cardiac dysfunction. Many patients with DCMmrEF are asymptomatic. Use of HF medical therapy in asymptomatic individuals with mild systolic dysfunction remains a topic of debate due to lack of evidence from clinical trials. Additional markers of HF risk are desirable.

Myocardial deformation, measured by left ventricular (LV) global longitudinal strain (GLS), offers prognostic utility beyond LV ejection fraction (LVEF) in patients with non‐ischaemic HF with significant systolic dysfunction.^6^ LV GLS may be a more sensitive measure of mild contractile dysfunction than LVEF and thus may have a role in identifying patients at risk of progressive HF.^7^ However, an association between strain and ventricular arrhythmia has additionally been reported.^8^ LV GLS is also associated with adverse cardiovascular outcomes in healthy population cohorts and relatives of patients with DCM.^9^, ^10^, ^11^ The prognostic value of strain has not been specifically studied in patients with DCMmrEF. Thus, we sought to evaluate whether LV GLS, derived from CMR feature‐tracking, and LGE could be used to predict (i) progressive HF and (ii) SCD in patients with DCMmrEF, to determine whether routine measurement of strain is worthwhile in this population for prognostic purposes. We hypothesized that LV GLS would predict incident HF events among patients with DCMmrEF, and when combined with LGE sequences, that multiparametric CMR could be used to predict both HF events and SCD. Notably, there are limited long‐term outcome data available describing the natural history of patients with DCMmrEF. Hence the identification of novel prognostic markers in this population could aid in identifying the subset at highest risk of deterioration who may benefit from enhanced disease surveillance and early treatment escalation.

## Methods

### Study population

Consecutive patients referred for a CMR between 2009 and 2017 from our clinical service and a network of surrounding hospitals were prospectively enrolled into the Royal Brompton Hospital Cardiovascular Research Centre (RBH CRC) Biobank. The study complied with the Declaration of Helsinki and was approved by the National Research Ethics Service (South Central Hampshire B Research Ethics Committee, Reference 19/SC/0257). All participants provided written consent. The inclusion criterion was DCMmrEF, defined as increased indexed LV end‐diastolic volume and LVEF that was ≥40% but lower than age‐ and sex‐adjusted nomograms at the point of enrolment.^12^ Exclusion criteria were significant ischaemic heart disease (IHD) (defined as stenosis >50% in a major epicardial coronary artery, inducible ischaemia on functional testing or prior coronary revascularization), adverse loading conditions (uncontrolled hypertension or severe primary valve disease), congenital heart disease, active myocarditis, or an alternative cardiomyopathy. Patients with DCMmrEF were further classified as either (i) index DCMmrEF, where patients had not previously had a LVEF recorded as <40%; or (ii) recovered DCMmrEF in cases where patients had a previously recorded LVEF <40% which had then improved to LVEF ≥40% on the study enrolment CMR.

### Cardiovascular magnetic resonance

All patients underwent a CMR scan at 1.5 Tesla (Sonata/Avanto, Siemens, Erlangen, Germany). Breath‐hold steady‐state free precession sequences were performed to produce long and short‐axis cine images. Gadopentetate dimeglumine or gadobutrol (0.1 mmol/kg) was injected intravenously and an inversion recovery gradient echo sequence undertaken to acquire the LGE images at 10 min. Left and right ventricular volumes and LV mass were measured using CMRtools (Cardiovascular Imaging Solutions, London, UK) and indexed to body surface area. Cine images were analysed for two‐dimensional LV GLS using Medis Qstrain (v2.0) and QMass (v8.1) on Medis Suite v3.1 (Medis Medical Imaging Systems, Leiden, The Netherlands) by a single expert operator blinded to clinical outcomes. This involved semi‐automatic delineation of the LV endocardial borders in the three long‐axis views (3‐chamber, 4‐chamber and 2‐chamber) with manual adjustment. LV contours were tracked via the Qstrain package and GLS calculated automatically as the average of the three long‐axis peak strain values derived from strain curves (online supplementary *Figure* *S1*). LGE presence was assessed by two independent expert CMR readers, with a third adjudicating cases of disagreement. LGE was considered present when seen in both long‐ and short‐axis planes, in two orthogonal views, extending beyond the LV/RV insertion points.

### Follow‐up and endpoints

Clinical follow‐up data were obtained from primary care records, hospital medical records and postal questionnaires sent to patients. Updated follow‐up data were acquired approximately every 2–3 years after enrolment and curated by the study team into a centralized database. Survival status was ascertained from the National Health Service Spine. Death certificates were obtained from the UK General Register Office and autopsy reports were retrieved from either Coroners' Offices or hospitals. Follow‐up duration was measured from CMR date and truncated at 10 years. All events were adjudicated by a panel of experienced cardiologists who were blinded to all CMR data, using medical information, death certificates, autopsy reports and implantable cardioverter‐defibrillator (ICD) reports. All potential arrhythmic events were reviewed by a cardiologist with expertise in implantable cardiac devices; ICD electrograms were reviewed where necessary. Patients were censored at the time of first event. The primary endpoint was progressive HF, defined as a composite of HF hospitalization, LV assist device implantation, or HF death. HF hospitalization was defined as a hospital admission with a primary diagnosis of HF lasting at least 24 h, in which the patient reported worsening symptoms of HF and had objective evidence of worsening or new HF, and received initiation/intensification of HF‐specific treatment. The secondary endpoint was a composite of SCD or aborted SCD (aSCD). Aborted SCD was defined as either an appropriate ICD shock for a ventricular arrhythmia, or a non‐fatal episode of ventricular fibrillation or spontaneous sustained ventricular tachycardia causing haemodynamic compromise and requiring cardioversion (see online supplementary methods for full endpoint definitions).

### Statistical analysis

Patient characteristics are presented as frequencies (%) for categorical variables and median (interquartile range [IQR]) for continuous variables. Mann–Whitney test was used to compare continuous variables. Chi‐squared test was used to compare categorical variables. Ordinal values were compared using Cochran–Armitage test for trend. Correlation between measures of LV structure and function was assessed using Spearman rank correlation coefficient. Cumulative incidence curves were estimated using Kaplan–Meier method and compared using the log‐rank test. The association between patient characteristics and the primary endpoint was examined using univariable and multivariable Cox proportional hazard modelling. A multivariable model was built for the primary endpoint using a backward stepwise selection method of candidate variables with an entry criterion of *p* < 0.05. The basis for this method of variable selection for the multivariable model related to the lack of other studies available in the literature describing predictors of HF events in patients with DCMmrEF, hence a stepwise selection represented the most data‐driven approach. The authors considered that an alternative method of *a priori* variable selection using variables conventionally associated with adverse HF outcomes in more severe DCM phenotypes may not be applicable in this population of patients with mild disease. The optimal LV GLS threshold was derived from Youden index in receiver operating characteristic analyses. Model performance was assessed using Harrell's C‐statistic. A two‐tailed *p*‐value of <0.05 was considered statistically significant. Statistical analyses were conducted on Rstudio (v4.2.2): *survival* and *survminer* packages were used for survival analysis; figures were generated using *ggplot* package.

## Results

### Cohort

Of 696 patients with a confirmed diagnosis of DCM, 355 patients met the inclusion criteria for DCMmrEF and were included in the study. Of these, 214 (60.3%) had index DCMmrEF and 141 (39.7%) had recovered DCMmrEF. Significant IHD was excluded by invasive coronary angiography in 213 patients (60.0%), computed tomography coronary angiography in 30 patients (8.5%), and functional test (stress perfusion CMR, nuclear scan, or stress echocardiogram) in 54 patients (15.2%). The remaining 59 patients had a very low clinical probability of IHD and did not undergo dedicated investigation to formally exclude: of these, 30 patients were aged ≤40 years, none had prior angina, and none required revascularization or experienced an acute coronary syndrome during follow‐up.

A higher proportion of the cohort were men (216 patients [60.8%]) and most were Caucasian (312 patients [87.9%]). The median age was 54 years (IQR 43–64). The median LVEF was 49% (46–54), the median LV GLS was −18.1% (−20.0 to −15.1) and 32% of the cohort had LGE on CMR (*Table* *1*). There was no difference in age, sex, ethnicity or New York Heart Association (NYHA) class between index and recovered DCMmrEF subgroups. Patients with index DCMmrEF had marginally higher LVEF and less abnormal LV GLS compared to recovered DCMmrEF, and a greater proportion of patients with recovered DCMmrEF were treated with HF drug therapies compared to patients with index DCMmrEF (online supplementary *Table* *S1*). LV GLS was moderately correlated with LVEF (*r* = −0.57) and weakly correlated with LV volumes (online supplementary *Figure* *S2*).

**Table 1:** Baseline characteristics of patients with dilated cardiomyopathy with mildly reduced ejection fraction classified by median left ventricular global longitudinal strain

	Overall (*n* = 355)	LV GLS ≤ −18.1% (*n* = 178)	LV GLS > −18.1% (*n* = 177)	*p*‐value
Demographics				
Age, years	54 (43–64)	52 (42–63)	56 (43–65)	0.050
Female sex	139 (39.2)	74 (41.6)	65 (36.7)	0.408
Caucasian	312 (87.9)	157 (88.2)	155 (87.6)	0.984
Past medical history				
Hypertension	106 (29.9)	47 (26.4)	59 (33.3)	0.19
Diabetes mellitus	30 (8.5)	9 (5.1)	21 (11.9)	**0.034**
Current smoker	33 (9.3)	15 (8.4)	18 (10.2)	0.319
History of atrial fibrillation	57 (16.1)	22 (12.4)	35 (19.8)	0.086
History of chemotherapy	18 (5.1)	3 (1.7)	15 (8.5)	**0.006**
Family history of SCD	62 (17.5)	38 (21.3)	24 (13.6)	0.097
Family history of DCM	69 (19.4)	44 (24.7)	25 (14.1)	**0.027**
NYHA class				
I	201 (56.6)	103 (57.9)	98 (55.4)	0.299
II	115 (32.4)	60 (33.7)	55 (31.1)	
III/IV	39 (11)	15 (8.4)	24 (13.6)	
Medication				
ACEi/ARB	280 (78.9)	137 (77.0)	143 (80.8)	0.447
Beta blocker	217 (61.1)	110 (61.8)	107 (60.5)	0.576
MRA	80 (22.5)	30 (16.9)	50 (28.2)	**0.016**
CMR characteristics				
LVEDVi, ml/m^2^	106 (90–120)	105 (97–117)	108 (96–121)	0.387
LVESVi, ml/m^2^	54 (45–62)	50 (43–57)	57 (49–65)	**<0.001**
LVEF, %	49 (46–54)	53 (49–56)	47 (43–50)	**<0.001**
RVEDVi, ml/m^2^	84 (72–99)	87 (74–101)	82 (70–95)	**0.009**
RVESVi, ml/m^2^	36 (27–45)	37 (27–46)	35 (36–44)	0.210
RVEF, %	58 (53–65)	58 (53–64)	58 (51–65)	0.596
LAVi, ml/m^2^	51 (42–62)	51 (42–61)	52 (42–64)	0.418
LGE presence	112 (31.5)	54 (30.3)	58 (32.8)	0.077

*Note*: Bold indicates significant values (*p* < 0.05).

Data are presented as median (interquartile range), or *n* (%).

ACEi, angiotensin‐converting enzyme inhibitor; ARB, angiotensin II receptor blocker; CMR, cardiovascular magnetic resonance; DCM, dilated cardiomyopathy; LAVi, left atrial volume index; LGE, late gadolinium enhancement; LVEDVi, left ventricular end‐diastolic volume index; LVEF, left ventricular ejection fraction; LVESVi, left ventricular end‐systolic volume index; LV GLS, left ventricular global longitudinal strain; MRA, mineralocorticoid receptor antagonist; NYHA, New York Heart Association; RVEDVi, right ventricular end‐diastolic volume index; RVEF, right ventricular ejection fraction; RVESVi, right ventricular end‐systolic volume index; SCD, sudden cardiac death.

### Primary endpoint

**Figure 1:**
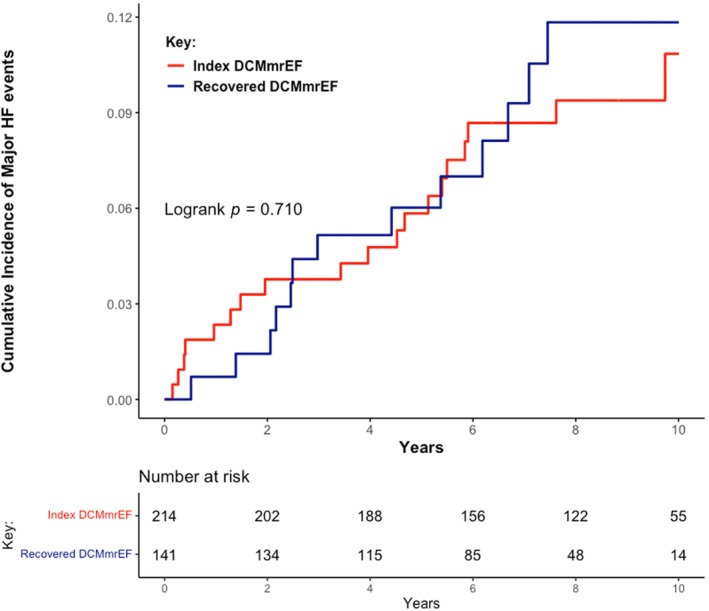
Cumulative incidence of major heart failure (HF) events classified by index dilated cardiomyopathy with mildly reduced ejection fraction (DCMmrEF) versus recovered DCMmrEF.

**Figure 2:**
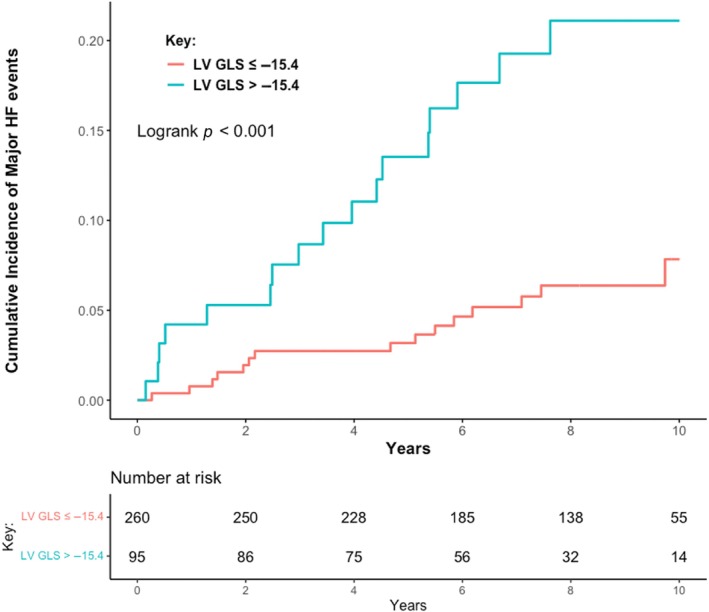
Cumulative incidence of heart failure (HF) events among patients with dilated cardiomyopathy with mildly reduced ejection fraction classified by left ventricular global longitudinal strain (LV GLS) −15.4%.

Over a median follow‐up of 7.8 years (IQR 5.2–9.4), 32 patients (9%) met the primary endpoint of progressive HF. This included 19/214 (8.9%) with index DCMmrEF and 13/141 (9.2%) with recovered DCMmrEF; there was no difference in the cumulative incidence of HF events between patients with index and recovered DCMmrEF (*p* = 0.710; *Figure* *1*). In total, 28 patients met the endpoint due to HF hospitalization, four due to HF death and none from LV assist device implantation. On univariable analysis of all patients with DCMmrEF, LV GLS was associated with the primary endpoint when considered as a continuous variable (per % hazard ratio [HR] 1.15, 95% confidence interval [CI] 1.04–1.26, *p* = 0.004), whereas LVEF was not (per 10% HR 0.64, 95% CI 0.33–1.22, *p* = 0.172). On multivariable analysis, LV GLS remained associated with the primary endpoint (per % HR 1.10, 95% CI 1.00–1.21, *p* = 0.045), alongside left atrial volume index (LAVi) (per 10 ml/m^2^ HR 1.08, 95% CI 1.02–1.13, *p* = 0.005) and NYHA class III/IV (HR 3.77, 95% CI 1.49–9.57, p = 0.005) (online supplementary *Table* *S2*). The optimal threshold for dichotomizing LV GLS was −15.4% (online supplementary *Figure* *S3*). Patients with LV GLS > −15.4% had a higher cumulative incidence of the primary endpoint than those with LV GLS ≤ −15.4% (log‐rank *p* < 0.001) (*Figure* *2*) (*Graphical Abstract*). The 5‐year HF event rate was 13.5% for patients with LV GLS > −15.4%, compared to a 5‐year HF event rate of just 3.2% in patients with LV GLS ≤ −15.4%. On univariable analysis, LV GLS > −15.4% was associated with the primary endpoint (HR 3.45, 95% CI 1.72–6.92, *p* < 0.001). On multivariable analysis, GLS > −15.4% remained associated with the primary endpoint (HR 2.70, 95% CI 1.30–5.94, *p* = 0.008) (*Table* *2*). The C‐statistic for the multivariable model containing LV GLS > −15.4%, NYHA class and LAVi was 0.764; when LV GLS was removed from this model the C‐statistic dropped to 0.707. LGE presence was not associated with the primary endpoint (HR 1.49, 95% CI 0.73–3.01, *p* = 0.270). Addition of LGE to the model containing LV GLS > −15.4%, NYHA class and LAVi did not improve model precision (C‐statistic remained 0.764).

**Table 2:** Univariable and multivariable association between baseline and cardiovascular magnetic resonance characteristics of all patients with dilated cardiomyopathy with mildly reduced ejection fraction and the primary endpoint

Characteristic	Univariable		Multivariable	
	HR (95% CI)	*p*‐value	HR (95% CI)	*p*‐value
Age, per 10 years	1.39 (1.08–1.81)	**0.012**		
Male sex	0.78 (0.39–1.58)	0.496		
Hypertension	1.62 (0.80–3.29)	0.179		
Diabetes mellitus	2.76 (1.13–6.71)	**0.025**		
Current smoker	0.92 (0.28–3.01)	0.886		
History of AF	1.89 (0.86–4.15)	0.112		
NYHA class II	1.85 (0.80–4.28)	0.148		
NYHA class III/IV	5.37 (2.28–12.7)	**<0.001**	3.87 (1.55–9.70)	**0.004**
LVEDVi, per 10 ml/m^2^	1.01 (1.00–1.03)	0.121		
LVESVi, per 10 ml/m^2^	1.02 (1.00–1.05)	0.066		
LVEF, per 10%	0.64 (0.33–1.22)	0.172		
LV GLS > −15.4%	3.45 (1.72–6.92)	**<0.001**	2.70 (1.30–5.94)	**0.008**
RVEDVi, per 10 ml/m^2^	1.09 (0.92–1.28)	0.341		
RVESVi, per 10 ml/m^2^	1.22 (0.98–1.52)	0.080		
RVEF, per 10%	0.77 (0.51–1.14)	0.189		
LAVi, per 10 ml/m^2^	1.12 (1.07–1.18)	**<0.001**	1.06 (1.01–1.12)	**0.025**
LGE presence	1.49 (0.73–3.01)	0.270		

*Note*: Bold indicates significant values (*p* < 0.05).

Left ventricular global longitudinal strain is included as a dichotomized variable.

AF, atrial fibrillation; CI, confidence interval; HR, hazard ratio; LAVi, left atrial volume index; LGE, late gadolinium enhancement; LVEDVi, left ventricular end‐diastolic volume index; LVEF, left ventricular ejection fraction; LVESVi, left ventricular end‐systolic volume index; LV GLS, left ventricular global longitudinal strain; NYHA, New York Heart Association; RVEDVi, right ventricular end‐diastolic volume index; RVESVi, right ventricular end‐systolic volume index; RVEF, right ventricular ejection fraction.

### Secondary endpoint

**Figure 3:**
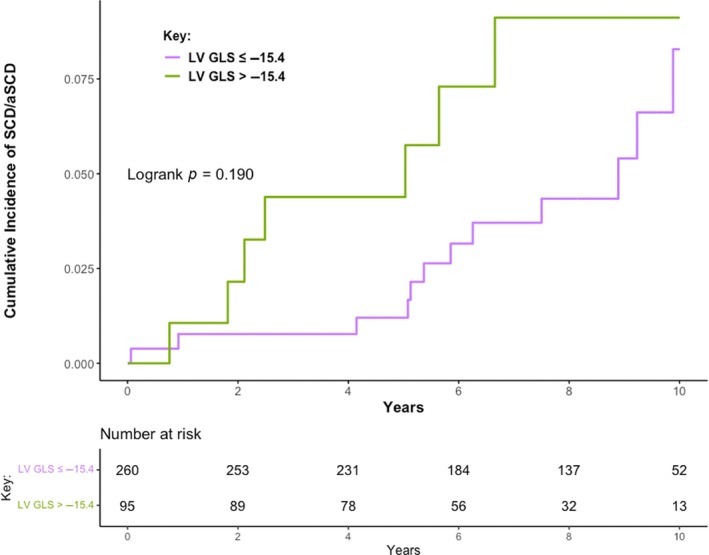
Cumulative incidence of sudden cardiac death (SCD) or aborted SCD (aSCD) among patients with dilated cardiomyopathy with mildly reduced ejection fraction classified by left ventricular global longitudinal strain (LV GLS) −15.4%.

In total, 19 patients (5.4%) met the SCD composite endpoint during follow‐up, including 12/214 (5.6%) with index DCMmrEF and 7/141 (5.0%) with recovered DCMmrEF. This was due to appropriate shocks from ICDs implanted during follow‐up in nine patients, resuscitated ventricular fibrillation/tachycardia cardiac arrests requiring cardioversion/defibrillation in six patients who had not had ICDs implanted, and SCD in four patients. There was no difference in the cumulative incidence of SCD/aSCD between patients with index and recovered DCMmrEF (log‐rank *p* = 0.890; online supplementary *Figure* *S4*). In total, 50 (14.1%) patients with DCMmrEF underwent ICD/cardiac resynchronization therapy‐defibrillator (CRT‐D) implantation during follow‐up, of whom 29 (13.6%) had index DCMmrEF and 21 (14.9%) had recovered DCMmrEF. Six patients had ICD/CRT‐D devices implanted for secondary prevention (five following resuscitated cardiac arrest occurring during follow‐up, one following resuscitated cardiac arrest occurring prior to enrolment with device implantation after enrolment). LV GLS was not associated with SCD/aSCD on univariable analysis (per % HR 1.07, 95% CI 0.95–1.22, *p* = 0.257). There was no difference in the cumulative incidence of SCD/aSCD when LV GLS was dichotomized (log‐rank *p* = 0.190; *Figure* *3*). LVEF was also not associated with SCD/aSCD (per 10% HR 1.00, 95% CI 0.44–2.29, *p* = 0.997). The only variable associated with SCD/aSCD on univariable analysis was LGE presence (LGE+) (HR 3.58, 95% CI 1.39–9.23, *p* = 0.008) (online supplementary *Table* *S3*). Accordingly, patients with LGE+ DCMmrEF had a higher cumulative incidence of SCD/aSCD than those with no LGE (LGE−) (log‐rank *p* = 0.005) (online supplementary *Figure* *S5*). The 5‐year SCD/aSCD event rate was 3.7% for patients with LGE+ DCMmrEF, compared to 1.3% for LGE− DCMmrEF.

### Predictive value of global longitudinal strain and late gadolinium enhancement in index versus recovered dilated cardiomyopathy with mildly reduced ejection fraction

Left ventricular GLS > −15.4% was associated with a higher cumulative incidence of progressive HF in the subgroup with index DCMmrEF (log‐rank *p* < 0.001); a similar trend was observed in the subgroup with recovered DCMmrEF, although this did not meet statistical significance, likely due to the smaller subgroup sample size and lower number of events (online supplementary *Figure* *S6*).

## Discussion

### Left ventricular global longitudinal strain is associated with progressive heart failure in patients with dilated cardiomyopathy with mildly reduced ejection fraction

The major finding from this study was that LV GLS was associated with the primary endpoint of progressive HF in this cohort of patients with DCMmrEF after accounting for other important covariates, and regardless of the trajectory of LVEF. Classifying patients using a LV GLS threshold of −15.4% identified a subset of patients with DCMmrEF with a near three‐fold enhanced risk of progressive HF. The inclusion of LV GLS in a multivariable model enabled more accurate prediction of HF events. By contrast, LVEF was not associated with HF events in this population. These data suggest that routine strain analysis using feature‐tracking may be a worthwhile add‐on to standard CMR analysis in this population to inform prognosis. We also demonstrate that NYHA class and LAVi are other important predictors of HF events in this patient group, as is observed in larger cohorts of patients with more severe phenotypes.^13^

### Left ventricular global longitudinal strain is not associated with sudden cardiac death

In contrast to the primary endpoint, LV GLS was not associated with the secondary endpoint of SCD or aSCD. The only parameter that predicted SCD/aSCD was the presence of LGE, in keeping with findings from a separate earlier cohort study undertaken at our institution.^5^ This finding is consistent with observation elsewhere that myocardial fibrosis, as detected by LGE, represents the major arrhythmic substrate for ventricular arrhythmia in DCM. The biological mechanisms that underpin this association relate to fixed and/or functional electrical conduction block occurring at the border between normal myocardium and regions of fibrosis, which propagate re‐entry circuits leading to ventricular arrhythmia.^14^, ^15^

### Clinical application of cardiovascular magnetic resonance for precision risk prediction in dilated cardiomyopathy with mildly reduced ejection fraction

The evidence base for treating patients with HF with mildly reduced LVEF largely exists in those with symptoms/elevated N‐terminal pro‐B‐type natriuretic peptide.^16^, ^17^ Many patients with DCMmrEF however do not have symptoms (including 57% of this cohort), and may have normal natriuretic peptides. The data presented in this paper raise the question of whether LV GLS, derived from feature‐tracking CMR, could be used as an imaging biomarker to identify those at highest risk of progressive HF that may obtain most benefit from early initiation and aggressive up‐titration of drug therapies; further data are required to test this at scale. It is additionally recognized that some patients with DCMmrEF are at significant risk of SCD yet remain ineligible for primary prevention ICD implantation based on LVEF.^14^, ^18^ ICDs may be considered in patients with DCM and LVEF >35% in the presence of additional risk factors, such as high‐risk genotypes.^18^ The data presented in this paper suggest a potential further role of CMR to enhance ICD decision making among such patients. As LGE predicted SCD but not progressive HF, and LV GLS predicted progressive HF but not SCD, it may be feasible to combine these metrics to identify patients with higher arrhythmic risk (LGE+) without concomitantly enhanced competing risk of progressive HF (LV GLS ≤ −15.4%), potentially identifying a subgroup of patients most likely to derive a survival benefit from their implantation. The prognostic value of LV GLS in patients with DCMmrEF is incremental to conventional measures of cardiac structure and function, such as LVEF. This finding differs from our observation that left atrial strain did not confer additive prognostic information beyond left atrial volumes and left atrial emptying fraction.^19^ Importantly, patients with DCMmrEF represent a neglected subpopulation for whom there are limited data to aid in prognostication and guide therapy. We propose that studies such as this are of particular value to build further evidence to inform future clinical guidelines to improve management of these patients. Whilst external validation of the prognostic value of LV GLS is required, it is important to note that this technique does not require any additional CMR sequences or image acquisition and measurement is partially automated, easy to learn and quick to perform. LV GLS may therefore represent a cost‐effective adjunct to current risk stratification tools in this population.

### What this study adds to the literature

The clinical determinants of progressive HF have not previously been reported among patients with DCMmrEF. Our results support a role for feature‐tracking CMR analysis in this population to enhance precision prognostication. The finding that LGE was the sole determinant of SCD/aSCD validates previous work from our group in an earlier cohort and emphasizes the robustness of LGE for arrhythmic risk stratification in this population.^5^

### Limitations

Patients in this study were enrolled from a single UK referral centre and its hospital network and the study inclusion criteria required a clinical referral by a physician for a CMR, introducing a possible referral bias. It is possible that some events were missed in patients undergoing clinical follow‐up at hospitals outside our own, despite extensive attempts made to retrieve all relevant clinical follow‐up information for subjects in this study. The cohort was predominantly Caucasian and male and there was a low burden of comorbidity. These features of the cohort may limit generalizability of our findings. It is also unknown whether the findings observed are equally applicable to patients with different dominant pathological drivers of DCM or indeed patients with different underlying genetic substrate. Vendor‐specific differences in LV GLS measurement exist and further comparative data are required to test the association and prognostic threshold derived from this cohort using different CMR feature‐tracking analysis software.^20^ LV GLS can also be obtained from echocardiography using speckle‐tracking, and further work should focus on whether a similar association exists from strain parameters acquired from different imaging modalities. Cardiac biomarkers, including natriuretic peptides, were not routinely measured at the point of enrolment in this cohort and hence it remains unknown whether their inclusion in multivariable models may interact with the association between LV GLS and the primary endpoint. Exploring whether LV GLS and LGE are associated with natriuretic peptide levels would also have been of interest but was not possible. T1 data were not obtained as part of the CMR protocol and may have further enhanced risk stratification in the cohort. Finally, the low number of events is a limitation of this study and precluded further analysis in the subgroups of patients with index and recovered DCMmrEF. Future work will focus on validating our findings in larger multicentre external cohorts and integrating blood biomarkers and genetic data with the aim of further enhancing risk prediction in this population.

## Conclusion

Among patients with DCMmrEF, LV GLS stratifies the risk of progressive HF and LGE stratifies the risk of SCD, regardless of LVEF trajectory. By contrast, LVEF did not predict either HF events or SCD. The results of this study support routine CMR evaluation of patients with DCMmrEF to identify those at greatest risk of future pump failure or sudden death among this highly heterogeneous patient population.

## Supporting information


**Appendix S1.** Supporting Information.
