# Supplementary material for: Precision prediction of heart failure events in patients with dilated cardiomyopathy and mildly reduced ejection fraction using multi‐parametric cardiovascular magnetic resonance
Source: Eur J Heart Fail. 2024 Aug 15;26(12):2553–62. doi: 10.1002/ejhf.3425 (PMC7616534; doi:10.1002/ejhf.3425)
Supplement: Supplementary file 1 — Appendix S1. Supporting Information. [file EJHF-26-2553-s001.docx]

**Hammersley DJ et al.**

**Precision prediction of heart failure events in patients with dilated cardiomyopathy and mildly reduced ejection fraction using multi-parametric cardiovascular magnetic resonance**

**Supplementary Material**

[Supplementary Methods 2](#_Toc168088318)

[Supplementary Tables 4](#_Toc168088319)

[Supplementary Figure 8](#_Toc168088320)

[Supplementary Material References 14](#_Toc168088321)

# Supplementary Methods

*Clinical endpoint definitions*

The following definitions were used by adjudicators for the purpose of adjudicating clinical endpoints in this study.^1,2^

*CV Death: Sudden Cardiac Death*

Death that occurs unexpectedly and not within 30 d of an acute MI. Sudden cardiac death includes the following scenarios:

1. Death witnessed and occurring without new or worsening symptoms.
2. Death witnessed within 60 min of the onset of new or worsening cardiac symptoms unless the symptoms suggest acute MI.
3. Death witnessed and attributed to an identified arrhythmia (e.g., captured on an electrocardiographic recording, witnessed on a monitor, or unwitnessed but found on ICD review).
4. Death after unsuccessful resuscitation from cardiac arrest (e.g., ICD unresponsive sudden cardiac death, pulseless electrical activity arrest).
5. Death after successful resuscitation from cardiac arrest and without identification of a specific cardiac or noncardiac aetiology.
6. Unwitnessed death in a subject seen alive and clinically stable ≤24 h before being found dead without any evidence supporting a specific non-cardiovascular cause of death (information about the patient’s clinical status preceding death should be provided if available)

Unless additional information suggests an alternate specific cause of death (e.g., Death due to Other Cardiovascular Causes), if a patient is seen alive ≤24 h before being found dead, sudden cardiac death (criterion [f]) should be recorded.

*CV Death: HF*

Death associated with clinically worsening symptoms and/or signs of HF, regardless of HF aetiology.

*HF Hospitalisation*

An event where the patient is admitted to the hospital where each of the following criteria apply:

1. Primary diagnosis of HF.
2. Length of stay is at least 24 h (or extends over a calendar date if the hospital admission and discharge times are unavailable).
3. The patient exhibits new or worsening symptoms of HF on presentation and objective evidence of new or worsening HF.
4. Receives initiation or intensification of treatment specifically for HF.

*Aborted SCD*

Aborted SCD diagnosed if patients have received an appropriate implantable cardioverter-defibrillator (ICD) shock for ventricular arrhythmia, or had a nonfatal episode of ventricular fibrillation or spontaneous sustained ventricular tachycardia causing hemodynamic compromise and requiring cardioversion.

# Supplementary Tables

**Supplementary Table 1: Baseline characteristics of patients with dilated cardiomyopathy with mildly reduced left ventricular ejection fraction classified by index versus recovered disease subgroups**

|  | **Total (N=355)** | **Index DCMmrEF (N=214)** | **Recovered DCMmrEF (N=141)** | **P-value** |
| --- | --- | --- | --- | --- |
| ***Demographics*** |  |  |  |  |
| **Age** | 54 (43-64) | 53 (42-64) | 56 (44-64) | 0.174 |
| **Male** | 216 (60.8%) | 126 (58.9%) | 90 (63.8%) | 0.410 |
| **Caucasian** | 312 (87.9%) | 190 (88.8%) | 122 (86.5%) | 0.637 |
| ***Past Medical History*** |  |  |  |  |
| **Hypertension** | 106 (29.9%) | 65 (30.4%) | 41 (29.1%) | 0.887 |
| **Type 2 Diabetes** | 30 (8.5%) | 14 (6.5%) | 16 (11.3%) | 0.162 |
| **Smoker** | 33 (9.3%) | 21 (9.8%) | 12 (8.5%) | 0.468 |
| **History of AF** | 57 (16.1%) | 28 (13.1%) | 29 (20.6%) | 0.114 |
| **Prior Chemotherapy** | 18 (5.1%) | 5 (2.3%) | 13 (9.2%) | **0.015** |
| **Family History of SCD** | 62 (17.5%) | 45 (21.0%) | 17 (12.1%) | 0.073 |
| **Family History of DCM** | 69 (19.4%) | 51 (23.8%) | 18 (12.8%) | **0.006** |
| ***NYHA Class*** |  |  |  |  |
| **1** | 201 (56.6%) | 129 (60.3%) | 72 (51.1%) | 0.225 |
| **2** | 115 (32.4%) | 64 (29.9%) | 51 (36.2%) |  |
| **3 & 4** | 39 (11.0%) | 21 (9.8%) | 18 (12.8%) |  |
| ***Medications*** |  |  |  |  |
| **ACEi/ARB** | 280 (78.9%) | 156 (72.9%) | 124 (87.9%) | **0.003** |
| **Beta Blocker** | 217 (61.1%) | 112 (52.3%) | 105 (74.5%) | **<0.001** |
| **MRA** | 80 (22.5%) | 35 (16.4%) | 45 (31.9%) | **0.002** |
| ***CMR Characteristics*** |  |  |  |  |
| **LVEDVi, ml/m^2^** | 106 (90-120) | 106 (96-121) | 105 (96-117) | 0.427 |
| **LVESVi, ml/m^2^** | 54 (45-62) | 53 (44-61) | 56 (48-62) | 0.142 |
| **LVEF, per %** | 49.0 (46-54) | 51 (47-54) | 48 (44-52) | **<0.001** |
| **LV GLS, per %** | -18 (-20--15) | -19 (-20--16) | -17 (-19--14) | **<0.001** |
| **LAVi, ml/m^2^** | 51 (42-62) | 52 (43-64) | 50 (42-61) | 0.218 |
| **RVEDVi, ml/m^2^** | 84.0 (72-99) | 85 (74-99) | 81 (70-97) | 0.162 |
| **RVESVi, ml/m^2^** | 36.0 (27-45) | 36 (27-45) | 35 (26-46) | 0.701 |
| **RVEF, ml/m^2^** | 58.0 (53-65) | 58 (54-64) | 59 (51-65) | 0.435 |
| **LGE presence** | 112 (31.5%) | 63 (29.4%) | 49 (34.8%) | 0.571 |
| Data presented as median (IQR) or n (%). ACEi = angiotensin-converting enzyme inhibitor; AF = atrial fibrillation; ARB = angiotensin II receptor blocker; DCM = dilated cardiomyopathy; LAVI = left atrial volume index; LGE = late gadolinium enhancement; LVEDVi = left ventricular end-diastolic volume index; LVEF = left ventricular ejection fraction; LVESVi = left ventricular end-systolic volume index; MRA = mineralocorticoid receptor antagonist; NYHA = New York Heart Association; RVEDVi = right ventricular end-diastolic volume index; RVEF = right ventricular ejection fraction; RVESVi = right ventricular end-systolic volume index. | | | | |

**Supplementary Table 2: Univariable and multivariable association between baseline and cardiovascular magnetic resonance characteristics of all patients with dilated cardiomyopathy with mildly reduced ejection fraction and the primary endpoint. Left ventricular global longitudinal strain is included as a continuous variable.**

|  | **Univariable** | | **Multivariable** | |
| --- | --- | --- | --- | --- |
| **Characteristic** | **HR** **(95% CI)** | **P** | **HR** **(95%CI)** | **P** |
| Age, per 10 years | **1.39** **(1.08-1.81)** | **0.012** |  | |
| Male | 0.78 (0.39-1.58) | 0.496 |  | |
| Hypertension | 1.62 (0.80-3.29) | 0.179 |  |  |
| Diabetes mellitus | **2.76 (1.13-6.71)** | **0.025** |  |  |
| Current smoker | 0.92 (0.28-3.01) | 0.886 |  |  |
| History of AF | 1.89 (0.86-4.15) | 0.112 |  |  |
| NYHA Class II | 1.85 (0.80-4.28) | 0.148 |  |  |
| NYHA Class III/IV | **5.37** **(2.28-12.7)** | **<0.001** | **3.77** **(1.49-9.57)** | **0.005** |
| LVEDVi, per 10ml/m^2^ | 1.01 (1.00-1.03) | 0.121 |  | |
| LVESVi, per 10ml/m^2^ | 1.02 (1.00-1.05) | 0.066 |  |  |
| LVEF, per 10% | 0.64 (0.33-1.22) | 0.172 |  |  |
| LV GLS , per % | **1.15 (1.04-1.26)** | **0.004** | **1.10 (1.00-1.21)** | **0.045** |
| RVEDVi, per 10ml/m^2^ | 1.09 (0.92-1.28) | 0.341 |  | |
| RVESVi, per 10ml/m^2^ | 1.22 (0.98-1.52) | 0.080 |  |  |
| RVEF, per 10% | 0.77 (0.51-1.14) | 0.189 |  |  |
| LAVi, per 10ml/m^2^ | **1.12** **(1.07-1.18)** | **<0.001** | **1.08** **(1.02-1.13)** | **0.005** |
| LGE presence | 1.49 (0.73-3.01) | 0.270 |  | |
| Data presented as hazard ratio (95% confidence interval). ACEi = angiotensin-converting enzyme inhibitor; AF = atrial fibrillation; ARB = angiotensin II receptor blocker; CI = confidence interval; HR = hazard ratio; LAVi = left atrial maximum volume index; LGE = late gadolinium enhancement; LVEDVi = indexed left ventricular end-diastolic volume; LVEF = left ventricular ejection fraction; LVESVi = indexed left ventricular end-systolic volume; LV GLS = left ventricular global longitudinal strain; MRA = mineralocorticoid receptor antagonist; NYHA = New York Heart Association; RVEDVi = indexed right ventricular end-diastolic volume; RVESVi = indexed right ventricular end-systolic volume; RVEF = right ventricular ejection fraction. | | | | |

**Supplementary Table 3: Univariable association between baseline and cardiovascular magnetic resonance characteristics of patients with dilated cardiomyopathy with mildly reduced ejection fraction and sudden cardiac death or aborted sudden cardiac death**

| **Characteristic** | **HR (95% CI)** | **P** |
| --- | --- | --- |
| Age, per 10 years | 1.05 (0.76-1.44) | 0.778 |
| Male | 3.34 (0.97-11.5) | 0.056 |
| Hypertension | 0.82 (0.29-2.27) | 0.699 |
| Diabetes mellitus | 0.64 (0.09-4.80) | 0.664 |
| Current smoker | 0.45 (0.06-3.39) | 0.439 |
| History of AF | 0.52 (0.12-2.28) | 0.385 |
| NYHA Class II | 0.56 (0.18-1.71) | 0.306 |
| NYHA Class III/IV | 0.88 (0.20-3.92) | 0.872 |
| ACEi/ARB | 0.97 (0.32-2.92) | 0.952 |
| Beta Blocker | 1.11 (0.44-2.81) | 0.830 |
| MRA | 0.46 (0.11-2.01) | 0.303 |
| LVEDVi, per 10ml/m^2^ | 1.07 (0.86-1.32) | 0.544 |
| LVESVi, per 10ml/m^2^ | 1.08 (0.78-1.49) | 0.651 |
| LVEF, per 10% | 1.00 (0.44-2.29) | 0.997 |
| LV GLS, per % | 1.07 (0.95-1.22) | 0.257 |
| RVEDVi, per 10ml/m^2^ | 1.14 (0.92-1.39) | 0.162 |
| RVESVi, per 10ml/m^2^ | 1.26 (0.96-1.66) | 0.091 |
| RVEF, per 10ml/m^2^ | 0.64 (0.64-1.06) | 0.082 |
| LAVi, per 10ml/m^2^ | 1.06 (0.96-1.18) | 0.262 |
| LGE | 3.58 (1.39-9.23) | **0.008** |
| Data presented as hazard ratio (95% confidence interval). AF = atrial fibrillation; CI = confidence interval; HR = hazard ratio; LAVi = left atrial maximum volume index; LBBB = left bundle branch block; LGE = late gadolinium enhancement; LVEDVi = indexed left ventricular end-diastolic volume; LVEF = left ventricular ejection fraction; LVESVi = indexed left ventricular end-systolic volume; LV GLS = left ventricular global longitudinal strain; NYHA = New York Heart Association; RVEDVi = indexed right ventricular end-diastolic volume; RVESVi = indexed right ventricular end-systolic volume; RVEF = right ventricular ejection fraction. | | |

# Supplementary Figure

**Supplementary Figure 1**: **Left ventricular global longitudinal strain was calculated using cardiovascular magnetic resonance feature-tracking.** GLS = global longitudinal strain; LV = left ventricular.

**
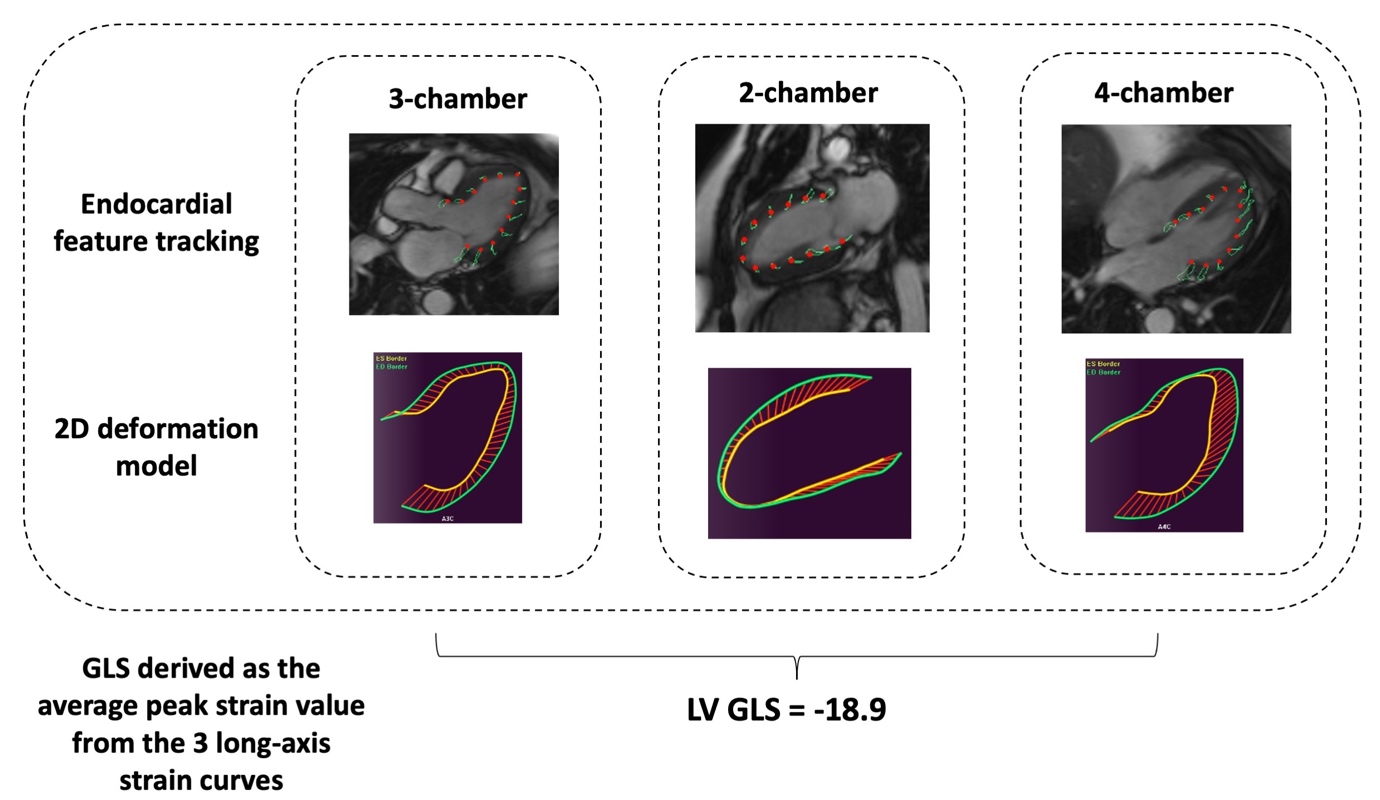
**

**Supplementary Figure 2**: **Correlation matrix for measures of left ventricular structure and function derived from cardiovascular magnetic resonance in a cohort of patients with dilated cardiomyopathy.** Correlation is depicted using colour scale for Pearson’s correlation coefficients. LVESVi = left ventricular end-diastolic volume index; LVEDVi = left ventricular end-diastolic volume index; LVEF = left ventricular ejection fraction; LV GLS = left ventricular global longitudinal strain.


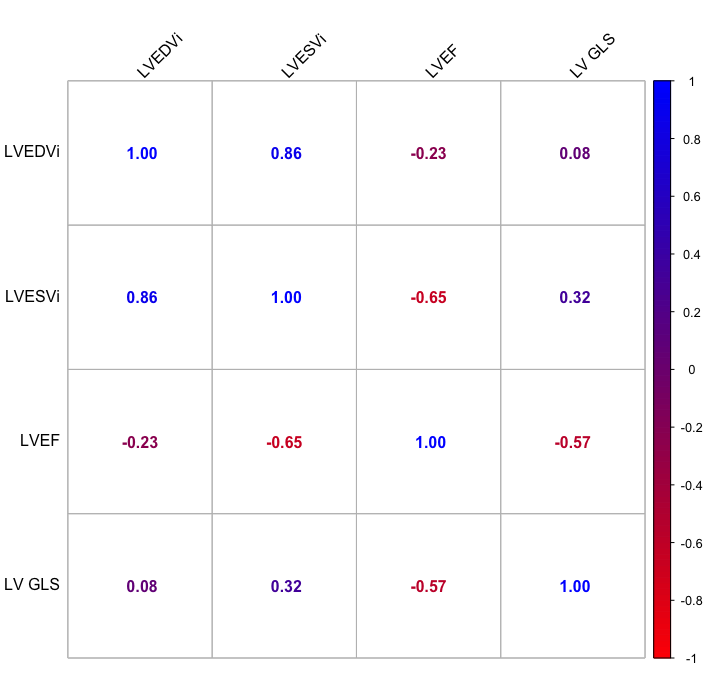


**Supplementary Figure 3: Receiver operator curve for the overall performance of left ventricular global longitudinal strain for the prediction of the primary endpoint of progressive heart failure:**


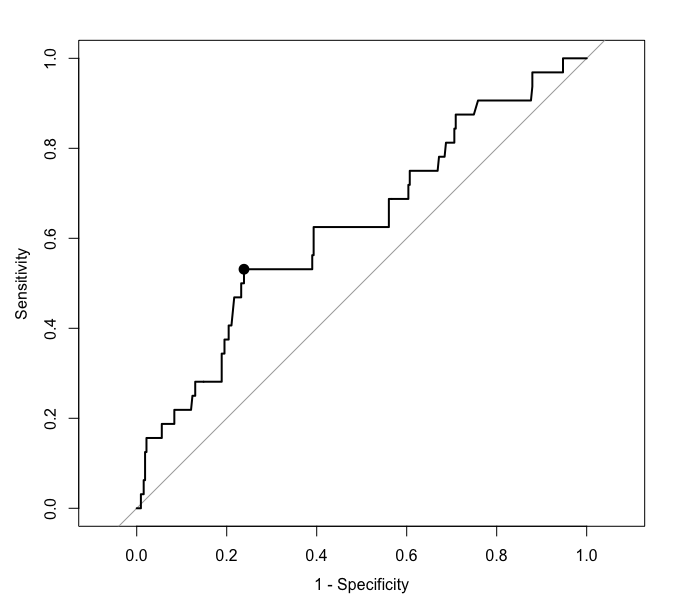


**Supplementary Figure 4: Cumulative incidence of the composite sudden cardiac death endpoint classified by index dilated cardiomyopathy with mildly reduced ejection fraction versus recovered dilated cardiomyopathy with mildly reduced ejection fraction.** aSCD = aborted sudden cardiac death; iDCMmrEF = index dilated cardiomyopathy with mildly reduced ejection fraction; rDCMmrEF = recovered dilated cardiomyopathy with mildly reduced ejection fraction; SCD = sudden cardiac death.

**
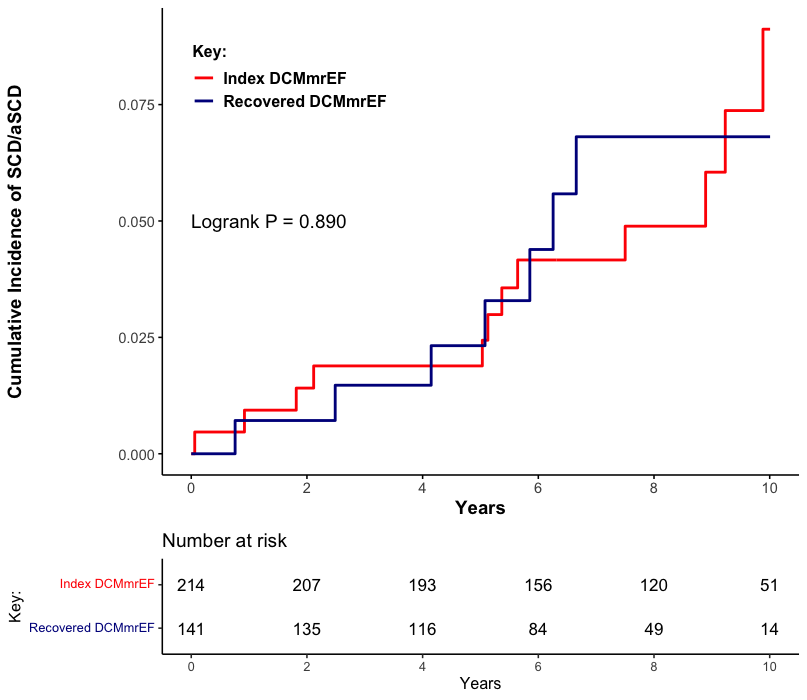
**

**Supplementary Figure 5: Cumulative incidence of sudden cardiac death or aborted sudden cardiac death in patients with dilated cardiomyopathy with mildly reduced ejection fraction classified by the presence of late gadolinium enhancement on cardiovascular magnetic resonance scan.** aSCD = aborted sudden cardiac death; LGE = late gadolinium enhancement; SCD = sudden cardiac death


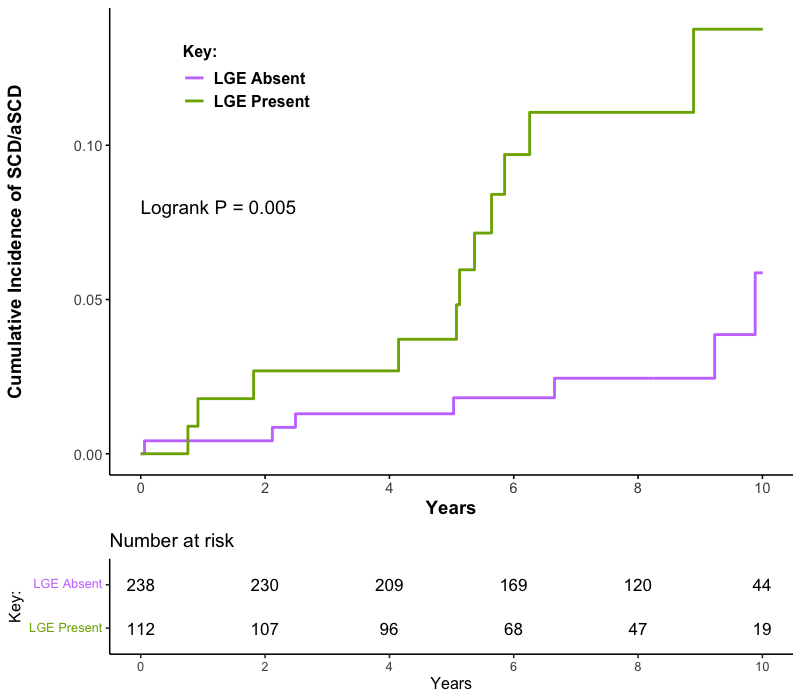


**Supplementary Figure 6: Cumulative incidence of major heart failure events classified by left ventricular global longitudinal strain in the subgroup with (A) index dilated cardiomyopathy with mildly reduced ejection fraction and (B) recovered dilated cardiomyopathy with mildly reduced ejection fraction.** HF = heart failure; LV GLS = left ventricular global longitudinal strain

**
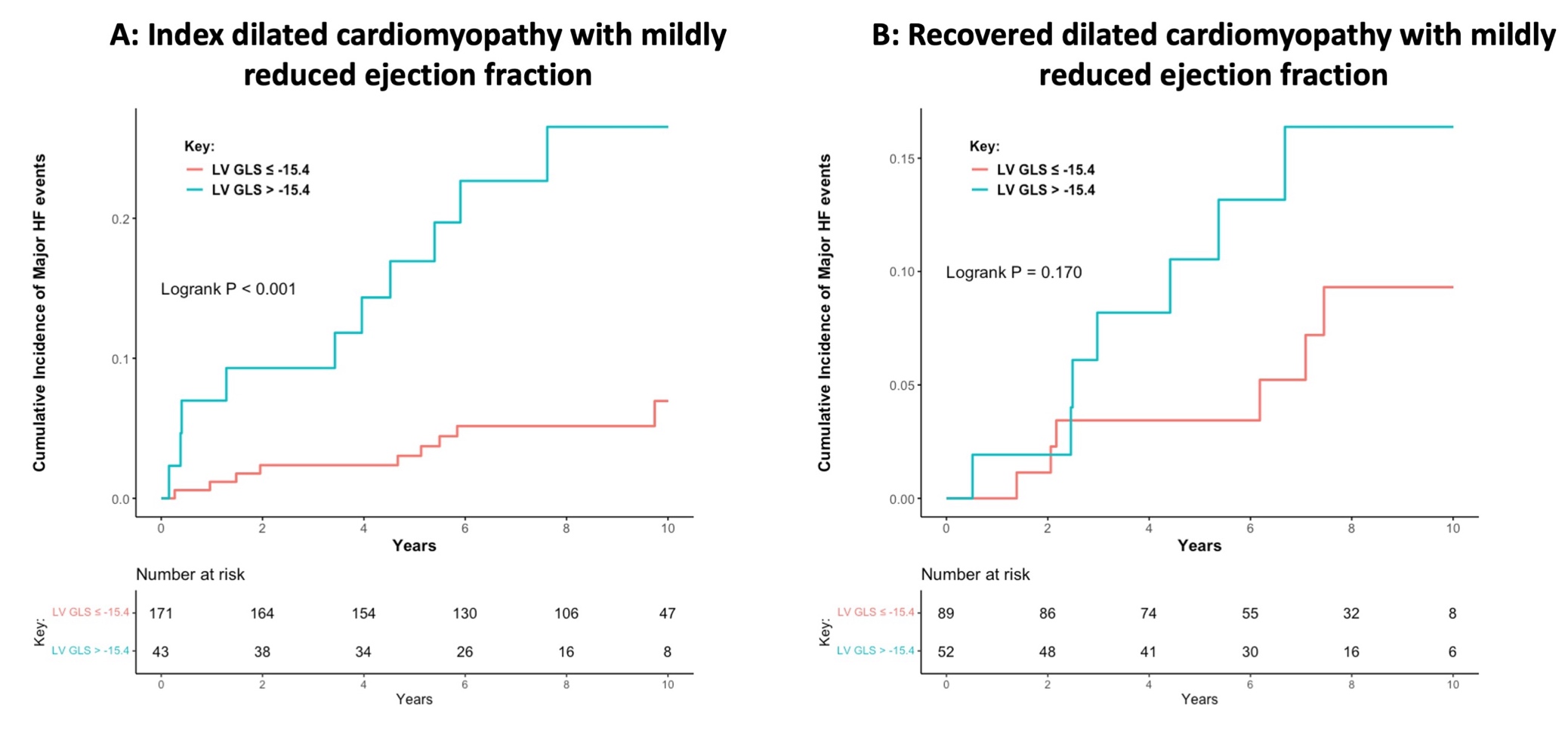
**

# Supplementary Material References

1. Hicks KA, Tcheng JE, Bozkurt B, et al. 2014 ACC/AHA key data elements and definitions for cardiovascular endpoint events in clinical trials: A Report of the American College of Cardiology/American Heart Association Task Force on Clinical Data Standards (Writing Committee to Develop Cardiovascu. *J Am Coll Cardiol* 2015;**66**:403–469.

2. American College of Cardiology/American Heart Association Task Force on Clinical Data Standards (ACC/AHA/HRS Writing Committee to Develop Data Standards on Electrophysiology), Buxton AE, Calkins H, et al. ACC/AHA/HRS 2006 key data elements and definitions for electrophysiological studies and procedures: a report of the American College of Cardiology/American Heart Association Task Force on Clinical Data Standards (ACC/AHA/HRS Writing Committee to Develop D. *Circulation* 2006;**114**:2534–2570.
